# Editorial: Gene Therapy for the Central and Peripheral Nervous System

**DOI:** 10.3389/fnmol.2018.00054

**Published:** 2018-02-22

**Authors:** Andrew P. Tosolini, George M. Smith

**Affiliations:** ^1^Sobell Department of Motor Neuroscience and Movement Disorders, Institute of Neurology, University College London, London, United Kingdom; ^2^Department of Neuroscience, Shriners Hospitals Pediatric Research Center, Lewis Katz School of Medicine, Temple University, Philadelphia, PA, United States

**Keywords:** gene therapy, CNS, PNS, AAV, lentivirus, non-viral vectors, neurons, glia

It is with great pleasure that we present the research topic dedicated to Gene Therapy for the Central (CNS) and Peripheral Nervous System (PNS). Gene therapy is at the cutting-edge of techniques utilized to develop novel therapeutics to treat insult(s) to the brain, spinal cord and/or peripheral nerves. Indeed, gene therapy can be applied via many different routes and as such can overcome the many obstacles facing oral and systemic delivery of synthetic drugs, thereby permit greater targeting of neural tissue. With this advantage, gene therapy has the potential to (1) correct disease-causing DNA mutations, (2) eliminate toxic RNA/proteins and/or (3) increase the expression of therapeutic proteins. Gene therapy can ameliorate aspects of debilitating neurological diseases and thus, can provide a platform for functional recovery.

Over the last few years, advances in basic science and technology have enabled enhanced pre-clinical strategies culminating in the emergence of sophisticated treatment options available for patients. The most recent gene therapy success is an FDA and EMA approved antisense oligonucleotide (ASO) that is the first and only treatment option available to treat spinal muscular atrophy. Moreover, ASOs have successfully reduced toxic protein levels in a phase 1/2a clinical trial to treat Huntington's Disease (HD). This treatment option was considered safe and well tolerated and was granted orphan drug designation by the FDA and EMA. These advances offer great hope to patients, their families, clinicians and basic scientists, and emphasizes the potential of gene therapy to treat “the incurable” neurological diseases/disorders.

In 2013, we launched this research topic to provide a platform to continue the discussion and amalgamate recent advances in gene therapy technology and strategies for the treatment of PNS and CNS disorders. In conclusion, we are proud to present an extremely productive discussion consisting of 18 articles in total comprised of eight original articles, five full-length reviews, three mini-reviews and one hypothesis and theory article and represents a world-wide collaboration with submissions received from USA, United Kingdom, Europe, Asia, New Zealand and Australia. This research topic discusses gene therapy strategies to treat neurological disorders including amyotrophic lateral sclerosis (ALS), spinal muscular atrophy (SMA), spinocerebellar ataxia (SCA), neuropathic pain, stroke, peripheral nerve injury and repair.

The novel studies presented in this research topic focus on improving transduction efficiency and gene transfer using adeno-associated virus (AAVs) and non-viral methods (Table 1). Tanguy et al compare the transduction efficiency between scAAV9 and AAVrh10 serotypes after systemic delivery. von Jonquieres et al. continue to demonstrate glial-specific transduction in the brain after injecting a chimeric AAV1/2 vector into neonatal striatum, despite using three differently sized MAG promoters. Jackson et al. combined an engineered AAV-serotype with a neuronal-specific promoter to increase transduction efficiency and reduce off-target effects after intravenous delivery. Wu et al. increase the intrinsic growth potential of injured sensory axons using combinatory treatment involving chondroitinase ABC and AAV-mediated constitutively active GTPase Rheb (Wu et al.).

**Table 1:** Highlights from the original research published in this research topic.

**Article**	**Highlights**	**Model**
Rogers et al.	Established a non-viral, antibody-based delivery method to transduce motor neurons *in vivo* after intraperitoneal injection. PEGylated polyethylenimine (PEI-PEG12) conjugated to a MRL2-antibody carrying DNA to the neurotrophin receptor p75 (p75NTR) targeted to motor neurons. 72 h after injection, ~25% of lumbar, ~18% or thoracic and 17% of cervical motor neurons were transduced.	Wild-type mice
Smolny et al.	Developed a non-viral, antibody-based delivery method for specific gene transfer in microglia *in vitro* and *in vivo*. OX42-immunoporter can bind plasmid DNA, and is trafficked to lysosomes in microglia via CD11b receptor-mediated internalization. OX42-immunogenes were specific to microglia and not astrocytes, but did not induce robust gene expression *in vitro* and *in vivo*.	*In vitro* and Wild-type mice
Tanguy et al.	Compared transduction efficiencies of scAAV9 and AAVrh10 in the brain, spinal, cord and peripheral nervous tissue after intravenous delivery in neonatal mice. AAVrh10 transduction was superior in the medulla, cerebellum, hippocampus, cortex, dorsal spinal cord, and spinal motor neurons. Dose-related transduction efficiency differences were observed in the sciatic nerve.	Wild-type mice
Jackson et al.	For the first time, AAV-PHP.B was demonstrated to transduce the rat CNS. After intravenous delivery in neonatal rats AAV-PHP.B was demonstrated to have a higher transduction efficiency than AAV9 when using the same CBA promoter. AAV-PHP.B with a synapsin promoter resulted in an enhanced transduction efficiency and neuronal specificity that induced TDP-43-like pathology and ALS-like phenotypes.	Wild-type rats
von Jonquieres et al.	Three MAG promoter sizes (0.3, 1.5, and 2.2 kb) were packaged into AAV-cy5 vector and were delivered into the striatum in wild-type neonates. All three promoter sizes exclusively transduced oligodendrocytes. Robust and oligodendrocyte-specific long-term GFP expression was reported at 8 months after neonatal delivery.	*In vitro* and Wild-type mice
Oliván et al.	Application of a non-toxic, tetanus toxin fragment (TTC) to spinal cord organotypic cultures increased SMN levels. Intramuscular injections of TTC reduced mRNA of autophagy markers (*Becn1, Atg5, LC3*, and *p62*) and pro-apoptotic genes (*Bax* and *Casp3*) in the spinal cord and downregulated *LC3* and *Casp3* expression in skeletal muscle in SMA mice. Intramuscular TTC application is suggested to show a compensatory effect in the expression of certain genes involved in muscle damage response, oxidative stress and calcium homeostasis in SMA mice.	*Ex vivo* and SMNΔ7 mice
Wu et al.	Intraganglionic injections of AAV5-caRHEB into cervical DRGs transduced mainly large caliber DRG neurons. ChABC treatment increased the number of regenerating axons through the DREZ irrespective of DRG-transduction, which resulted in sensory behavioral “responses.” caRHEB expression in DRGs after dorsal root crush enhances synaptic formation and/or functional regeneration into the spinal gray matter.	*In vitro* and Wild-type mice
Su et al.	miR-30b agomir transfection down-regulated the voltage-gated sodium channel Nav1.3 mRNA that was stimulated with TNF-α in primary DRG neurons. miR-30b overexpression reduced neuropathic pain after spinal nerve ligation, with demonstrated reduction in Nav1.3 mRNA and protein expression in both DRG neurons and spinal cord. miR-30b antagomir activated the Nav1.3 voltage-gated sodium channel.	*In vitro* and wild-type rats

In addition, Smolny et al. present a non-viral, antibody-based delivery method for microglia-specific gene transfer. Rogers et al. describe spinal motor neuron transduction after peripheral delivery of plasmid DNA as a PEGylated polyethylenimine conjugated antibody. Oliván et al. demonstrate that atoxic-tetanus neurotoxin fragment modifies expression of autophagy and pro-apoptotic genes in the spinal cord and skeletal muscle. Su et al. show miRNA-mediated suppression of specific voltage-gated sodium channels can alleviate neuropathic pain.

This research topic also includes a number of full-length and mini-reviews (Table 2). Murlidharan et al. describe the biology of different AAV strains, including their transduction profiles, cellular tropisms and mechanisms of CNS transport, for increased translational application. Parr-Brownlie et al. review lentiviral-vector approaches to enhance transduction efficiency, mediate cell-specificity, restrict gene expression spatially and temporally, and discuss limitations and future prospects. Tan et al. critically analyse the advantages and disadvantages of strategies using non-viral nucleic acids to deliver therapeutic genes by circumventing the immune response and thus, appeasing safety concerns potentially associated with viral-mediated gene therapies. Wagner et al. examine the gene and stem cell pre-clinical therapeutic options that preserve Purkinje cell health to treat SCA, and suggest that RNA interference (RNAi) might have great promise. Finally, Tosolini and Sleigh discuss important considerations learned from the success of a recently FDA- and EMA-approved gene therapy for SMA to develop viable gene therapies and strategies to treat ALS.

**Table 2:** Highlights from the review, mini-review and hypothesis and theory articles published in this research topic.

**Article**	**Highlights**	**Type**
Murlidharan et al.	Describes AAV-vector biology, their cellular entry mechanisms and axonal transport profiles of well-characterized AAV serotypes. Discusses the implications of AAV-vector applications (e.g., direct application, intravenous injections, etc.). Considers the safety aspects of AAV-mediated applications to the CNS.	Review
Parr-Brownlie et al.	Describes lentiviral vector biology, including modified envelope glycoproteins and the expression of transgenes under the regulation of cell-selective and inducible promoters. Deliberates on the benefit of lentiviral-vectors combined with other techniques such as anatomical tract-tracing, immunohistochemistry, confocal and electron microscopy. Proposes limitations and future perspectives including ways that lentiviral-vectors can contribute to the gene therapy clinical trials.	Review
Tan et al.	Explores the challenges facing non-viral nucleic acid delivery to the CNS and provides strategies to overcome them. Discusses the advantages and disadvantages of different administration routes of nucleic acid delivery. Considers how retrograde axonal transport can be used to deliver non-viral nucleic acids.	Review
Wagner et al.	Describes the epidemiology, molecular pathology and mouse models related to spinocerebellar ataxia-1 (SCA-1). Discusses the literature related to stem cell, gene and alternative therapies used to treat SCA-1. Identifies the various challenges for gene, stem cell and alternative therapies for SCA-1.	Review
Tosolini and Sleigh	Describes the epidemiology, genetics, classifications and mechanisms causing SMA and ALS/MND and deliberates on potential commonalities. Provides an update on clinical gene therapies for both SMA and ALS/MND. Identifies four key areas that ALS/MND gene therapies can learn from the recent success in the SMA gene therapies including therapeutic targeting, combinational treatment, considering the dose and drug concentration as well as optimizing the therapeutic timing.	Review
Craig and Housley	Provides a summary of the viral-mediated gene therapy research used to treat stroke. Highlights the key areas that gene therapy needs to address to ameliorate stroke including protein synthesis, delivery site and viral-vectors. Identifies therapeutic protein candidates for stroke treatment.	Mini-review
Stoica and Sena-Esteves	Summarizes the literature on AAV-mediated gene therapy studies that reduce SOD1 toxicity to treat SOD1-related ALS/MND. Discusses the current hurdles to be addressed to advance the development of clinical gene therapies such as non-cell autonomous toxicity, cellular and anatomical targeting and the delivery methods. Identifies RNA interference as a successful therapeutic target to ameliorate disease.	Mini-review
Yang et al.	Summarizes the development and application of the CRISPR/CAS9 toolkit. Describes the use of CRISPR/Cas9 to generate animal models of neurodegenerative diseases. Discusses how CRISPR/Cas9 can be applied to treat animal models of Parkinson's and Huntington's Disease.	Mini-review
Hoyng et al.	Summarizes the research on gene therapy in animal models of peripheral nerve repair and identify key future directions. Provides a perspective on the path for clinical translation for PNS-gene therapy for traumatic nerve injuries. Addresses efficacy and safety concerns for human applications and identify the ideal patient population for a proof-of-concept clinical study.	Hypothesis and theory

For the mini-reviews, Yang et al. discuss the “hot topic” of CRISPR/Cas9 gene editing and how these tools can be applied to various research models and the development of treatments for neurodegenerative disease, such as HD and Parkinson's disease. Craig and Housley focus on gene therapy approaches for stroke and discuss injury mechanisms, appropriate timings for therapeutic intervention and deliberate on candidate therapeutic proteins as therapeutic options. Stoica and Sena-Esteves deliver a succinct mini-review of AAV-mediated SOD1-ALS amelioration strategies and describe the hurdles to overcome for CNS gene delivery.

The Hypothesis and theory submission by Hoyng et al. summarize the state-of-research for peripheral nerve repair, identify future targets and provide a translational perspective on PNS gene therapy.

This research forum describes many important characteristics of diverse gene therapy applications for the development of tangible treatment options for different CNS and PNS disorders. Indeed, the advances in gene therapy strategies discussed within this research topic give hope that treatment options for many incurable CNS and PNS disorders are closer to becoming a viable clinical option.

## Author contribution

All authors listed have made a substantial, direct and intellectual contribution to the work, and approved it for publication.

### Conflict of interest statement

The authors declare that the research was conducted in the absence of any commercial or financial relationships that could be construed as a potential conflict of interest.

